# Supplementary material for: Anti-Neuroinflammatory Potential of a Nectandra angustifolia (Laurel Amarillo) Ethanolic Extract
Source: Antioxidants (Basel). 2023 Jan 19;12(2):232. doi: 10.3390/antiox12020232 (PMC9952224; doi:10.3390/antiox12020232)
Supplement: Supplementary file 1 [file antioxidants-12-00232-s001.zip › antioxidants-2024933-supplementary.pdf]

Figure S1

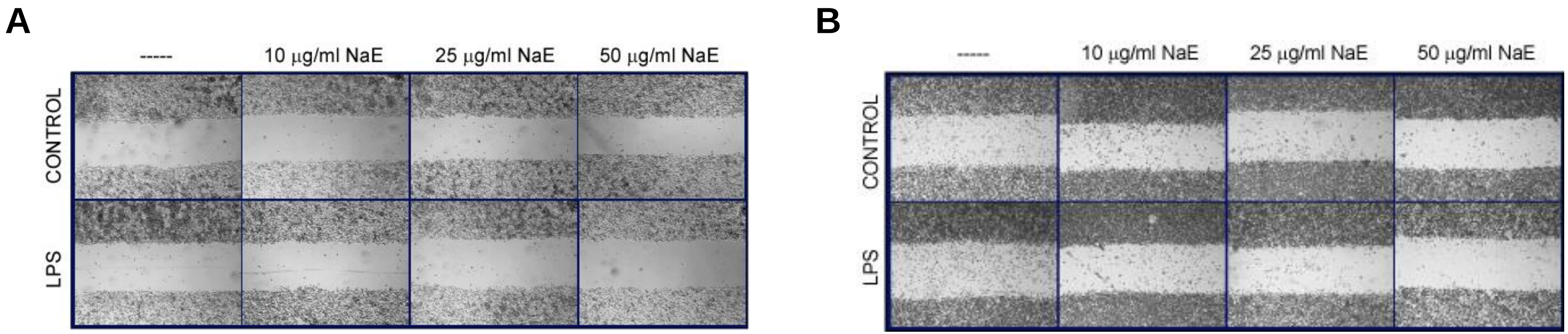

Figure S1. Effect of NaE extract on BV2 microglia functions. BV2 cells were pretreated for 60 min at 37 ºC with the indicated doses of NaE, and then stimulated or not with 0.1 µg/ml of LPS. Representative images showing: (A) Scratch at time 0; (B) Cell migration at 3 h. Magnification, 40×.

Figure S2

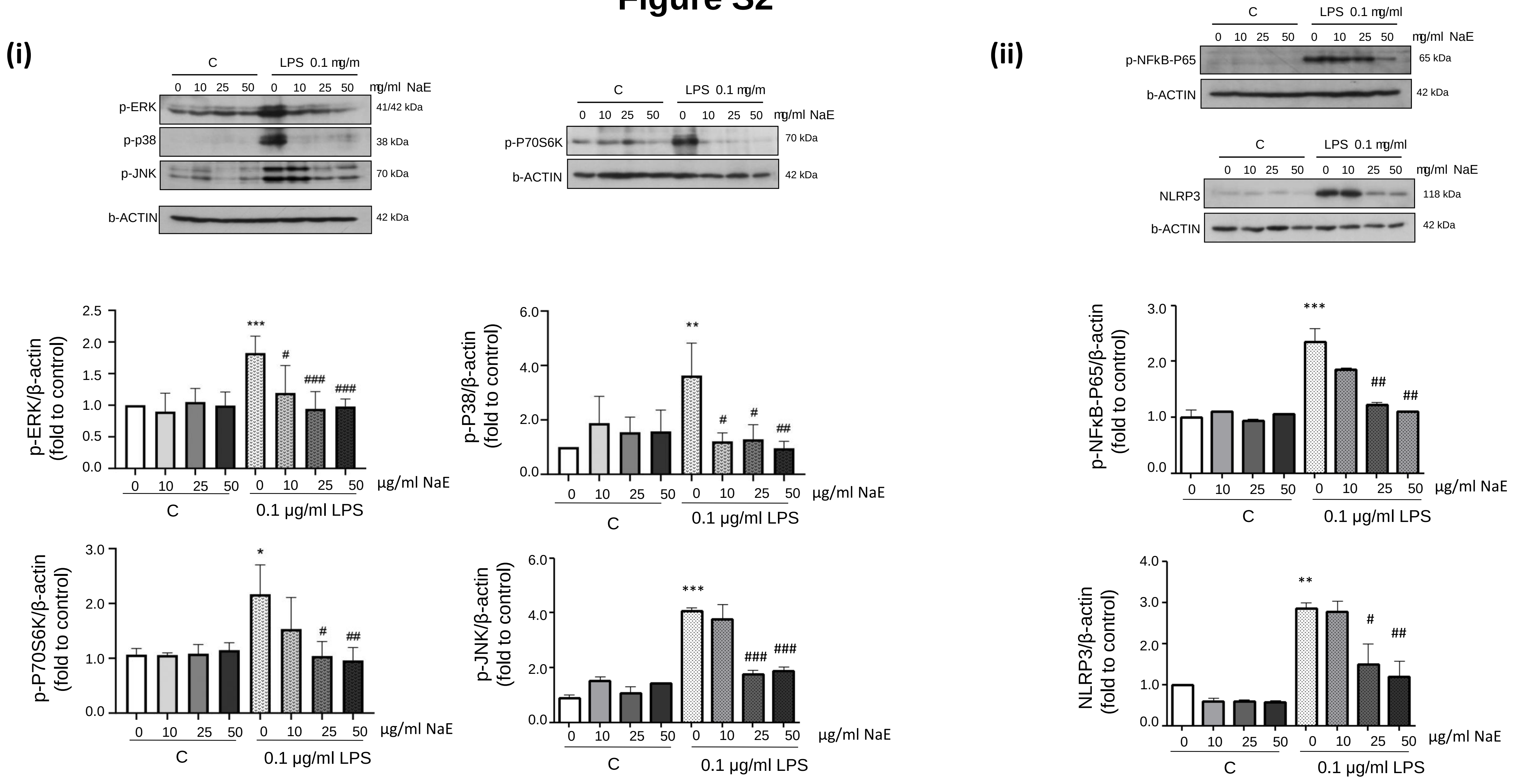

Figure S2. NaE treatment modulates BV2 microglial cells activation: Quantification. BV2 cells were pretreated for 60 min with the indicated doses of NaE. After 30 min (i) or 24 h (ii) of stimulation with 0.1 μg/ml of LPS, p-ERK, p-P38, p-JNK, p-NFKB-P65 and NLRP3 expression was identified in cell lysates by Western blot. Quantification graphs of the Western blots are shown with fold over the control in each condition (***$p<0.001$, **$p<0.01$, *$p<0.05$ *vs* control; ###$p<0.001$, ##$p<0.01$ and #$p<0.05$ *vs* LPS)

Figure S3

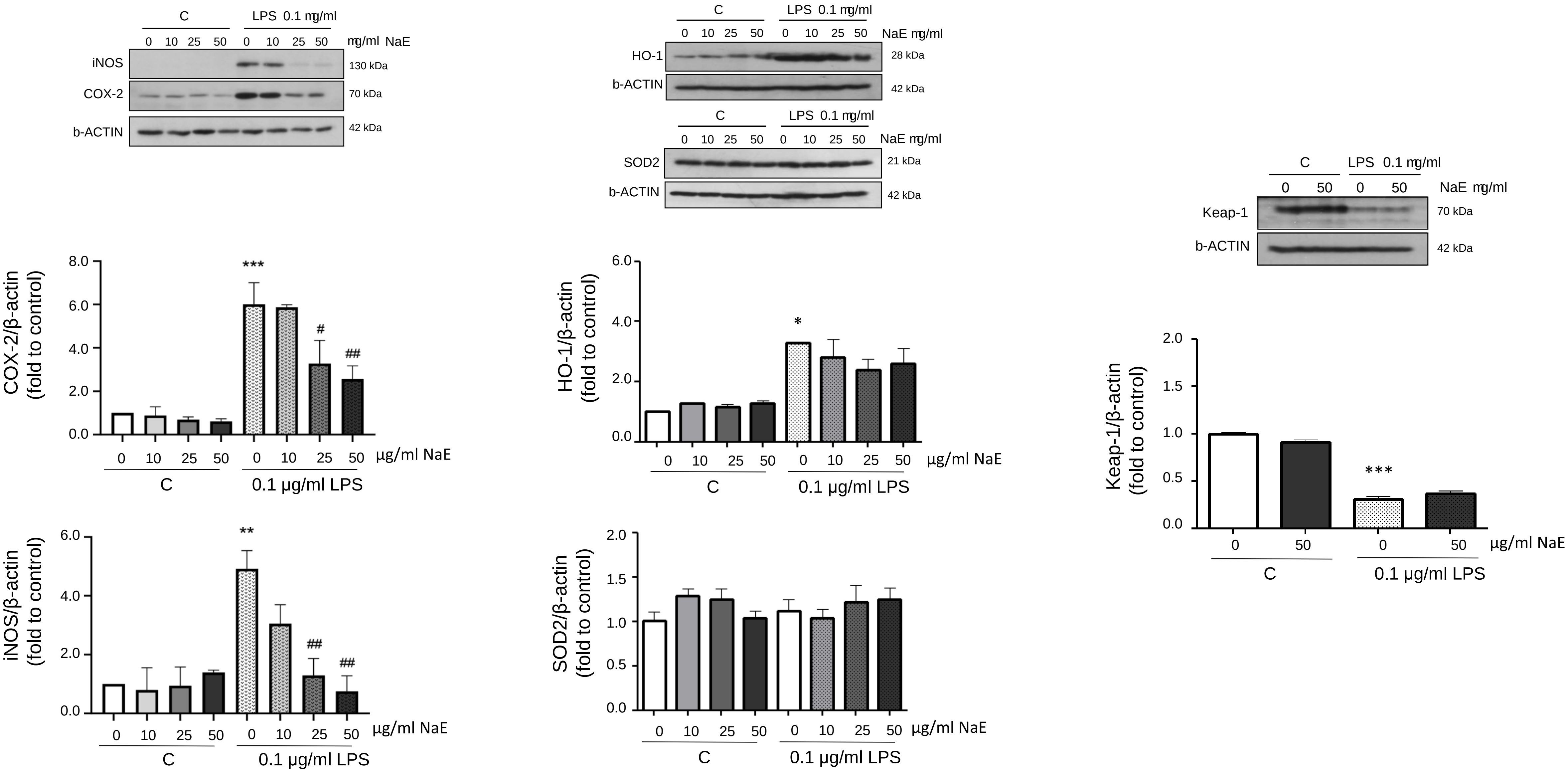

Figure S3. NaE treatment modulates BV2 microglial cells activation: Quantification. BV2 cells were pretreated for 60 min with the indicated doses of NaE. After 24 h of stimulation with 0.1 µg/ml of LPS, COX-2, iNOS, HO-1, SOD2 and Keap-1 expression was identified in cell lysates by Western blot. Quantification graphs of the Western blots are shown with fold over the control in each condition (***$p<0.001$, **$p<0.01$, *$p<0.05$ *vs* control; ##$p<0.01$ and #$p<0.05$ *vs* LPS)

Figure S4

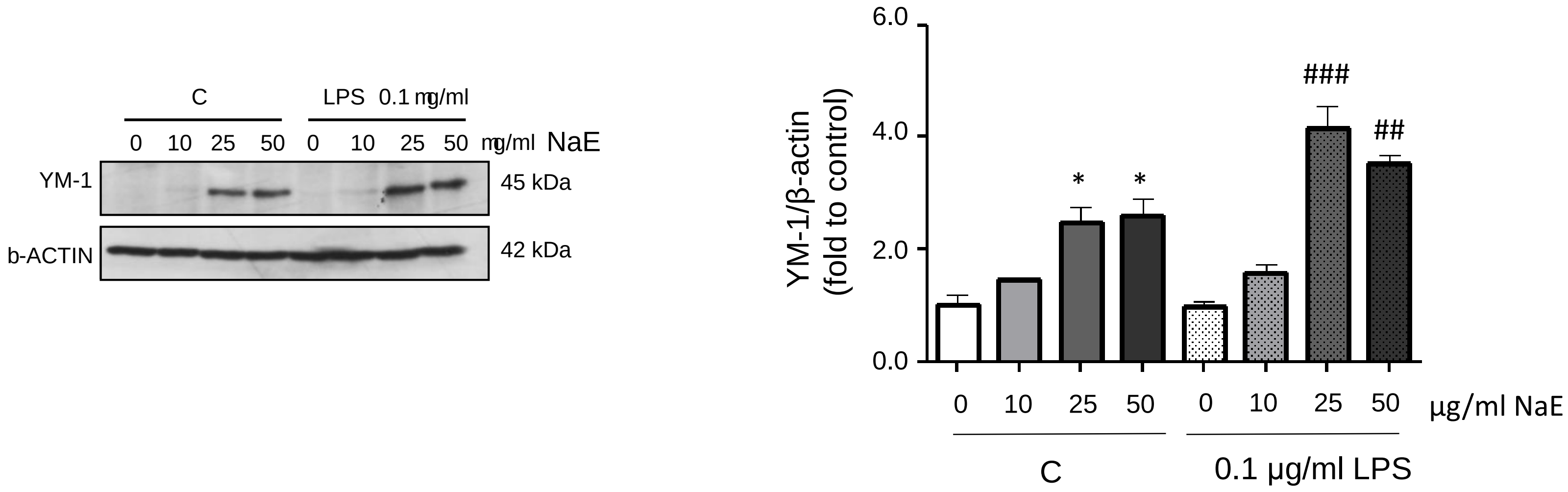

Figure S4. NaE treatment modulates BV2 microglial cells activation: Quantification. BV2 cells were pretreated for 60 min with the indicated doses of NaE. After 24 h of stimulation with 0.1 µg/ml of LPS, YM-1 expression was identified in cell lysates by Western blot. Quantification graphs of the Western blots are shown with fold over the control in each condition (*p<0.05 *vs* control; ###p<0.001 and ##p<0.01 *vs* LPS)

Table S1

Table S1. The concentration of the selected cytokines in the culture supernatants of BV2 cells pretreated for 60 min at 37 ºC with the indicated doses of NaE, and then stimulated with 0.1 μg/ml of LPS. Data presented as means ± SD (n = 3–5)

	CONTROL			LPS		
	0	NaE 25	NaE 50	0	NaE 25	NaE 50
TNFα (ng/ml)	0,325±0,110	0,346±0,170	0,342±0,110	75,713±1,960	17,562±6,870	7,070±1,830
IL-6 (ng/ml)	0,012±0,005	0,010±0,005	0,017±0,006	12,988±2,700	6,017±2,230	1,971±1,350
IL-1β (pg/ml)	10,060±3,027	12,690±1,707	15,978±2,784	75,880±3,719	10,811±1,349	14,212±1,609
IL-10 (pg/ml)	393,300±34,690	263,300±22,610	398,900±56,220	8,000±2,000	230,000±17,000	283,300±69,440
